# Within-person Variation In Emotional Exhaustion Among Caregivers For Older Adults

**DOI:** 10.1093/geroni/igab046.3752

**Published:** 2021-12-17

**Authors:** Tomoko Wakui

**Affiliations:** Tokyo Metropolitan Institute of Gerontology, Itabashi, Tokyo, Japan

## Abstract

Caregiving is everyday life for family members of older adults. Care recipients’ care requirements, service usage, and caregivers’ physical and emotional conditions differ day by day. Little is known how the differences and variances relate to informal caregivers’ mental health. This study aimed to examine informal caregivers’ day-to-day fluctuation in emotional exhaustion and discuss the within-person effects on mental health among informal caregivers. We developed the Caregiving Visualization Project toolkit (Care VIP), a software program for tracking on daily basis components of care experiences such as care task of Activity of Daily Living (ADL) and Instrumental Activity of Daily Living, service usages, and caregiving burden, as well as eight items of caregivers’ emotional exhaustion. We recruited study participants between May 2018 to March 2019 who provided instrumental help to community-dwelling older adults. A total of 75 participants, who accessed the Care VIP every day by using tablets or computers and completed a one-month study, were analyzed in this study. Females comprised 80.0%, and the average age of caregivers was 52.7 years (SD=9.1). The majority were those who provided care to parents (69.3%), and those who provided to parents-in-law and spouses were 16.0% and 5.3%, respectively. The average score of the eight items on the emotional exhaustion scale, with a 4-point Likert scale, was 23.4 (SD=4.9); however, each question showed different variations. Within-person effects on mental health among informal caregivers will be discussed.

